# Identification of Key Volatiles Differentiating Aromatic Rice Cultivars Using an Untargeted Metabolomics Approach

**DOI:** 10.3390/metabo11080528

**Published:** 2021-08-09

**Authors:** Yu Jie, Tianyu Shi, Zhongjei Zhang, Qiaojuan Yan

**Affiliations:** 1 College of Engineering, China Agricultural University, No. 17 Qinghua East Road Haidian District, Beijing 100083, China; jyu@ags.ac.cn; 2 Academy of National Food and Strategic Reserves Administration, No. 11 Baiwanzhuang Street, Beijing 100037, China; sty@ags.ac.cn

**Keywords:** aromatic rice, differential metabolites, acetoin

## Abstract

Non-aromatic rice is often sold at the price of aromatic rice to increase profits, seriously impairing consumer experience and brand credibility. The assessment of rice varieties origins in terms of their aroma traits is of great interest to protect consumers from fraud. To address this issue, the study identified differentially abundant metabolites between non-aromatic rice varieties and each of the three most popular aromatic rice varieties in the market using an untargeted metabolomics approach. The 656 metabolites of five rice grain varieties were determined by headspace solid-phase extraction gas chromatography-mass spectrometry, and the multivariate analyses were used to identify differences in metabolites among rice varieties. The metabolites most differentially abundant between Daohuaxiang 2 and non-aromatic rice included 2-acetyl-1-pyrroline and acetoin; the metabolites most differentially abundant between Meixiangzhan 2 and non-aromatic rice included acetoin and 2-methyloctylbenzene,; and the metabolites most differentially abundant between Yexiangyoulisi and non-aromatic rice included bicyclo[4.4.0]dec,1-ene-2-isopropyl-5-methyl-9-methylene and 2-methylfuran. Overall, acetoin was the metabolite that was most differentially abundant between the aromatic and non-aromatic rice. This study provides direct evidence of the outstanding advantages of aromatic rice and acts a reference for future rice authentication processes in the marketplace.

## 1. Introduction

China, which is the largest producer and consumer of rice worldwide, covers a vast area that includes widely different geographical and climatic conditions. These environmental differences have given rise to a rich variety of rice germplasms, including many rice varieties [1]. In general, rice is classified as either aromatic or non-aromatic depending on whether or not it is fragrant [2]. The fragrance of rice will affect its price and consumer acceptance. Aromatic rice *(Oryza sativa* L.) is a special rice species that can give off fragrance from their whole grain. Moreover, it still has aroma after cooking and is rich in amino acids, proteins and other nutrients, so it is highly favored by consumers around the world [3]. The aroma of aromatic rice is greatly influenced by geographical origin indication, and the economic return can be enhanced by the specific trait of the commodity in the specific producing area [4]. The most famous varieties of aromatic rice in China are produced in the Guangdong, Guangxi, Zhejiang, Liaoning, and Heilongjiang provinces [5]. Of these, Wuchang rice, originating from Wuchang in Heilongjiang province, is one of the most famous aromatic rice sold in the world [4]. Most aromatic rice varieties have substantially lower yields than non-aromatic rice varieties because aromatic rice varieties are less adaptable to changes in environmental conditions and are thus more affected by planting locality [3]. According to Chinanews, Wuchang produces only about 1.05 million tons of rice every year, but it is estimated that there are at least 10 million tons of Wuchang rice on the market [6]. Therefore, up to 90% of all Wuchang rice on the market must be counterfeit. Moreover, the price of aromatic rice is two or three times higher than that of non-aromatic rice [7]. To increase profits, inferior or non-aromatic rice is often mixed with aromatic rice and then labeled as premium quality rice [6]. This practice directly impacts consumers and impairs brand credibility [8]. In the past decades, the varieties of rice have been identified based on morphology (shape, width, and length), physiochemical properties such as amylose, starch, and protein content, cooking properties and eating properties [1,4]. However, most of the rice evaluation standards used at present are not graded strictly, i.e., the standard of ISO 7301–2011 and Codex Alimentarius Commission (CAC) [1]. It is thus critical to clarify the difference between aromatic rice and non-aromatic rice to ensure the authenticity of high-quality aromatic rice varieties [9]. Understanding the aroma traits of rice grain in different region will be of great benefit to the identification of geographical origin and the formulation of standards for aromatic rice in the future.

Rice aroma is greatly affected by the composition and proportion of volatile compounds in the rice grain [10]. Currently, more than 250 volatile compounds have been identified in rice. 2-Acetyl-1-pyrroline (2-AP) is a symbolic substance that distinguishes aromatic rice from non-aromatic rice [3]. It has a popcorn-like flavor and has been identified as the main compound that gives rice natural flavor [3]. Furthermore, hexanal, octanal, nonanal, (*E*)-2-octenal, decanal, 1-heptanol, and 1-octanol were identified as major aroma-active compounds in Jasmine rice [11]. Widiastuti et al. [12] using a dynamic headspace solid-phase extraction system coupled to a two-dimensional gas chromatography (GC × GC) coupled with a time-of-flight mass spectrometric detector (TOFMS) to distinguish the aromatic from non-aromatic rice grains. Fifty one kinds of volatile compounds were detected by this methods, and eight key-marker volatile compounds (i.e., pentanal, hexanal, 2-pentylfuran, 2,4-nonadienal, pyridine, 1-octen-3-ol and (*E*)-2-octenal) were selected for identifying the aromatic rice of Indonesia.

In addition to these, a significant proportion of rice varieties are composed of low molecular weight secondary metabolites, which are high vapor pressure volatile organic compounds (VOCs) synthesized during growth and development of the crop [4]. In recent years, VOC metabolomics have been used to identify the uniqueness and traceability of rice and to understand the properties of rice varieties [4]. Metabolomics, which is a promising and powerful tool for identifying qualitative differences in various biological systems, aims to thoroughly characterize low-molecule metabolites in organisms [13]. Headspace solid-phase extraction (HS-SPME) gas chromatography-mass spectrometry (GC-MS) accurately detects VOCs and has several advantages over other detection methods: relatively small sample sizes, no organic solvents, and reduced matrix effects [14]. HS-SPME-GC-MS can be used to extract high vapor pressure VOCs from rice grain by SPME fiber without pre-treatment.

The aim of this study was to identify potential VOC markers of aromatic rice and non-aromatic rice using an untargeted VOC metabolomics approach. Three aromatic rice varieties from well-known rice-producing areas in China were selected and compared with two non-aromatic rice varieties. VOC metabolites in the five rice varieties were detected, and the main VOC metabolites of each variety were identified. Finally, the metabolites associated with each of the five varieties were analyzed using multivariate analysis, and specific VOC metabolite markers for each aromatic rice variety were identified.

## 2. Results and Discussion

### 2.1. VOC Metabolites in Five Rice Varieties

Three well-known aromatic rice varieties from different rice-producing areas in China were chosen as research objects. These varieties are representative of each region and are all gold prize-winning, high-quality rice varieties [6]. HS-SPME-GC-MS was used to detect the VOC metabolites in all rice samples. A total of 656 VOC metabolites were identified and quantified. In a previous analysis of rice VOCs, Hu et al. [10] found that the aroma volatiles usually included an oxygen-containing group, a nitrogen group, a sulfur group, and an aromatic group. In this study, the identified species of VOC metabolites were mainly lipids, lipid-like compounds (i.e., hydrocarbons, alcohol and aldehyde), benzenoids, and organic oxygen compounds.

**Figure 1:**
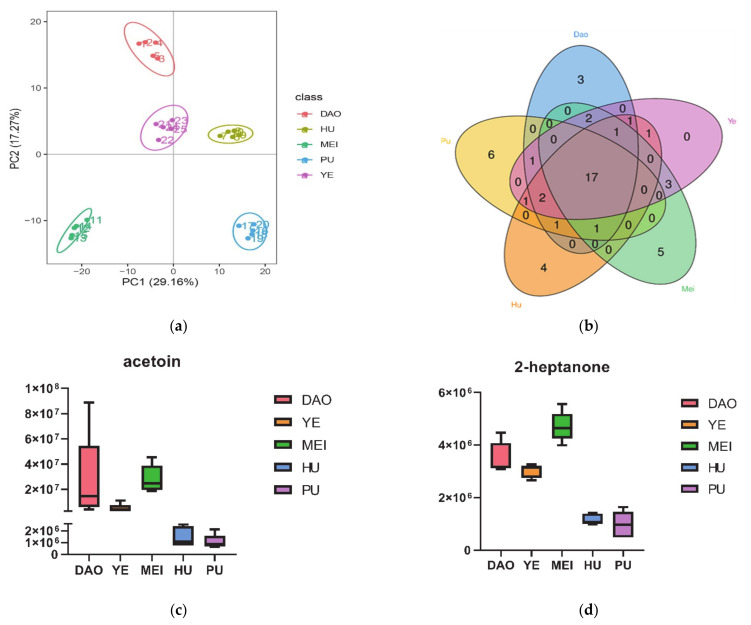
The differentiation of VOC metabolites in five rice varieties. (**a**) PCA analysis of five rice varieties. The PC1of *X*-axis and PC2 of *Y*-axis in the figure represent the scores of the first and second principal components respectively. The scattered points of different colors represent the samples of different rice varieties, and the ellipse is the 95% confidence interval. (**b**) Venn diagram of VOC metabolites in five rice varieties. (**c**) Box and whisker plots of the relative level of acetoin in five rice varieties. (**d**) Box and whisker plots of the relative level of 2-heptanone in five rice varieties. Dao stands for the variety of Daohuaxiang 2, Ye stands for the variety of Yexiangyoulisi, Mei stands for the variety of Meixiangzhan 2, Hu stands for the variety of Huanghuazhan, and Pu stands for the variety of Yanfeng 47.

To characterize the overall metabolic differences among the five rice varieties, as well as the variability among samples of each individual variety, the principal components of all samples were classified by similarity. The PCA of the five varieties is shown in Figure 1a. Two principal components cumulatively accounted for 46.36% of the total variation, with PC1 explaining 29.16% and PC2 explaining 17.2% of the variance. The replicate samples of each variety clustered together, forming five groups, but the five varieties were quite dissimilar. This shows that the growth environment of the rice variety, such as climate, soil conditions, and altitude, strongly influences the metabolite accumulation of the rice grain [10]. Indeed, previous authors have indicated that metabolite accumulation is substantially affected by environmental factors as well as by genetic factors [3].

To better understand the main substances that differed among the rice varieties, the 30 VOC metabolites with the highest abundances in each rice variety were selected for subsequent analysis. The selected VOC metabolites with their relative abundances across all the cultivars were illustrated in Table 1. A Venn diagram of these VOC metabolites showed that 17 metabolites were shared among all five rice varieties: 1-hexanol, fluoromethyloxirane, 1-butanol, 1-pentanol, dimethylsilanediol, acetone, acetic acid, hexanal, 1-octen-3-ol, 1-penten-3-ol, 2-pentylfuran, dibutyl phthalate, hexanoic acid, 1-heptanol, 2,4-dimethylbenzaldehyde, ethyl acetate, and decamethylcyclopentasiloxane (Figure 1b). These 17 metabolites were mainly alcohols and heterocyclic compounds. Alcohols in the subclass fatty alcohols, such as 1-hexanol, 1-butanol, 1-pentanol, 1-octen-3-ol, 1-penten-3-ol, and 1-heptanol, are the secondary products of polyunsaturated fatty acids and produce a soft smell [10]. Across all rice varieties, 1-octen-3-ol was the most abundant VOC; this compound produces an odor of mushrooms and straw [10]. Other abundant VOCs were 1-hexanol, contributing a grassy herbaceous and sweet flavor [15], and 1-butanol, which is produced via the degradation of aromatic compounds and has a floral smell [16]. As for heterocyclic compounds, such as furans, their primary pathways are associated with lipid oxidation or the Maillard reaction, and produce a caramel-like odor [10]. 2-Pentylfuran, which belongs to the furanone subclass and which has a nutty odor [17], was abundant in all rice grains. Finally, 2,4-dimethylbenzaldehyde is considered to be a vital aromatic compound in wild rice cultivars, with a mild, sweet, bitter-almond odor [18]. In contrast, Ch et al. [4] showed that alkanes, terpene, and alcohols were the major groups of VOCs in milled rice, which demonstrates the differences in VOC metabolites between milled rice and rice grains. Compared to milled rice, raw rice grains are alive and thus have more abundant metabolites and stronger aromas [19]. Rice bran accounts for 5–8% of the weight of the whole rice grain and has been used to extract oil in recent years [2]. Alcohols and phenols were the main volatiles in rice bran. 4-vinylguaiacol and 4-vinylphenol were reported associated with the aroma of rice bran and further contribute to the aroma of cooked rice and steam-distilled rice bran [2]. It was reported that unmilled black rice had more total volatiles than milled black rice [19]. Milling also substantially affects rice odor. As milling increases, raw rice flavor decreases, while glossiness, plumpness, and sweetness increase [20].

The abundances of five metabolites distinguished Meixiangzhan 2 from other rice varieties: 2,2-dichloroethanol, 3-methylbutanoic acid, pentanoic acid, nonanal, and 5-ethenyldihydro-5-methyl-2(3*H*)-furanone. 3-Methylbutanoic acid, a metabolite of butanone, is produced by the digestion of carbohydrates and proteins [16]; 5-ethenyldihydro-5-methyl-2(3*H*)-furanone gives off a vegetal odor [11]. The abundances of three VOC metabolites distinguished Daohuaxiang 2 from other rice varieties: 2-butanone, 2-acetyl-1-pyrroline and 1-methylcycloheptanol. Of these, 2-butanone is a common food-flavoring agent and is one of the markers that distinguish Indian rice from other rice varieties [4], and 2-acetyl-1-pyrroline (2-AP) is the main aromatic component of aromatic rice and contributes a popcorn-like aroma [21]. It is thought that 2-AP content mainly depends on varietal differences as well as the methods used for rice processing, storage, harvest, and evaluation [10]. According to the index, 1-methylcycloheptanol is an intermediate compound of flavors and fragrances that is commonly used as a food flavoring. N-butyl ether, indole, 1,2,4,5-tetramethylbenzene and 2-hexen-1-ol were the most important metabolites in Huanghuazhan rice, while Yanfeng 47 was distinguished from other rice varieties by the metabolites 3-isopropoxy-1,1,1,7,7,7-hexamethyl-3,5,5-tris(trimethylsiloxy)tetrasiloxane, decane,2,4,6-trimethyl, 2,6-dimethylnonane, 2-furanmethanol, 5-ethenyltetrahydro,5-trimethyl-2,2,4-trimethyl-1,3-pentanediol diisobutyrate and 3-octanol.

Overall, the abundances of two volatile metabolites distinguished the three aromatic rice varieties (Meixiangzhan 2, Daohuaxiang 2, and Yexiangyoulisi) from the two non-aromatic rice varieties (Huanghuazhan and Yanfeng 47): acetoin and 2-heptanone. Acetoin, also known as 3-hydroxy-2-butanone, is mainly synthesized by microorganisms and can also be produced in the cells of both mammals and plants such as rice and maize [22]. This compound, which has a pleasant yogurt odor and a creamy taste, is widely used in food flavorings, fragrances, and even biological pest controls [22]. The metabolite 2-heptanone contributes a fruity floral smell [2].

To visualize the differences between aromatic and non-aromatic rice, box and whisker plots were used to compare the relative levels of acetoin and 2-heptanone among the five rice varieties (Figure 1c,d). In previous studies of rice VOCs, 2-AP has been recognized as a marker of aromatic rice [3], but acetoin has rarely been reported. This may be related to the rice varieties studied: previous studies have mostly analyzed Thai or basmati rice [2], while the present study considered characteristic Chinese aromatic rice varieties exclusively.

### 2.2. Multivariate Analyses of Metabolites in the Five Rice Varieties

**Figure 2:**
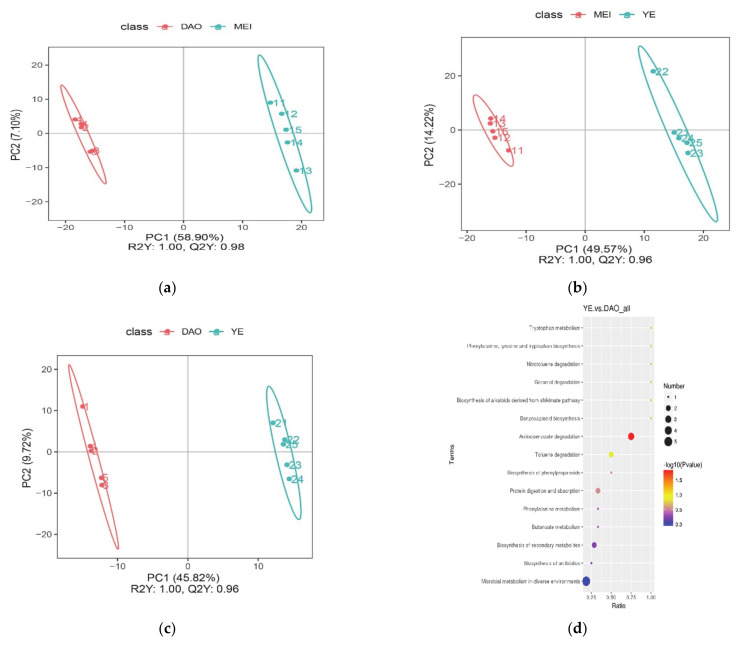

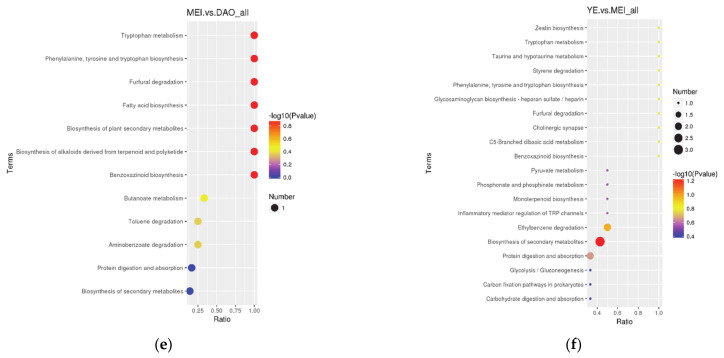
The VOC metabolic variation in aromatic rice varieties. (**a**) PLS-DA scores plot of Meixiangzhan 2 vs. Daohuaxiang 2. (**b**) PLS-DA scores plot of Yexiangyoulisi vs. Meixiangzhan 2. (**c**) PLS-DA scores plot of Yexiangyoulisi vs. Daohuaxiang 2. (**d**) The KEGG pathway that the significant differential metabolites take part in Yexiangyoulisi vs. Daohuaxiang 2. (**e**) The KEGG pathway that the significant differential metabolites take part in Meixiangzhan 2 vs. Daohuaxiang 2. (**f**) The KEGG pathway that the significant differential metabolites take part in Yexiangyoulisi vs. Meixiangzhan 2. In figures (**d**–**f**), the abscissa is x/y (i.e., the number of differential metabolites in the corresponding metabolic pathway divided by the total number of identified metabolites in the pathway). The higher the value on the abscissa, the higher the degree of differential metabolite enrichment in the corresponding pathway. Dot color represents the *p*-value of the hypergeometric test; smaller values reflect increased test reliability and greater statistical significance. The size of the dot represents the number of differential metabolites in the corresponding pathway; larger numbers indicate that more differential metabolites were identified in the corresponding pathway.

**Figure 3:**
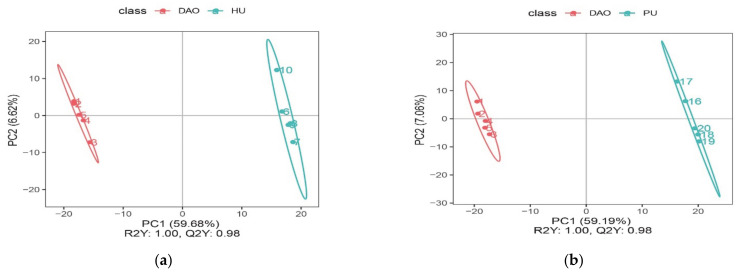

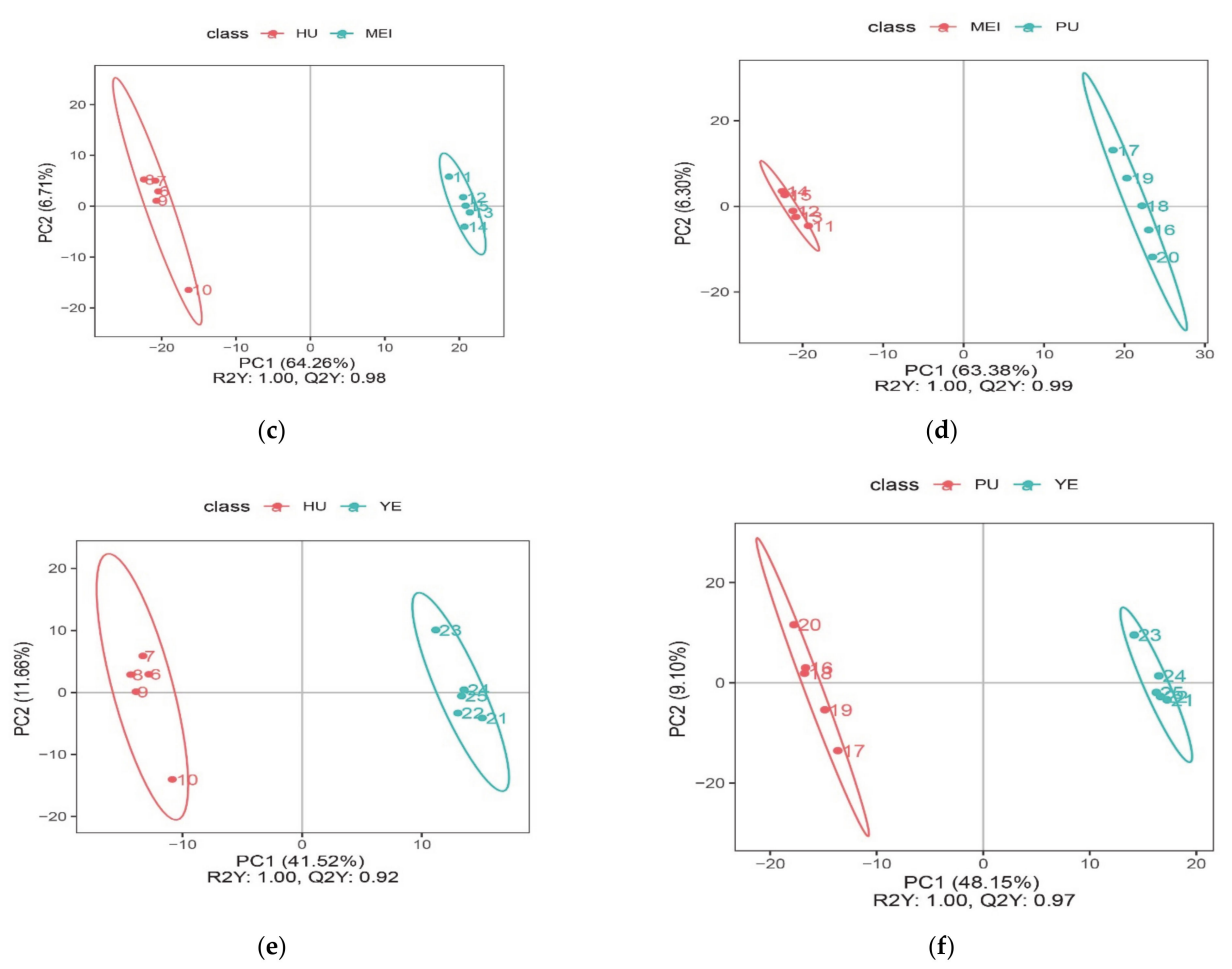
The VOC metabolic variation between aromatic and non-aromatic rice varieties. (**a**) PLS-DA scores plot of Daohuaxiang 2 vs. Huanghuazhan. (**b**) PLS-DA scores plot of Daohuaxiang 2 vs. Yanfeng 47. (**c**) PLS-DA scores plot of Meixiangzhan 2 vs. Huanghuazhan. (**d**) PLS-DA scores plot of Meixiangzhan 2 vs. Yanfeng 47. (**e**) PLS-DA scores plot of Yexiangyoulisi vs. Huanghuazhan. (**f**) PLS-DA scores plot of Yexiangyoulisi vs. Yanfeng 47.

PCAs were used to visualize the overall distributions of every pair of varieties, while PLS-DA, which is a supervised model, was used to maximally separate samples and to identify the maker metabolites. The PLS-DA was performed to develop a rice classification system based on differences in VOC metabolites that clearly discriminated between aromatic and non-aromatic varieties. Samples were tightly clustered by group, and groups were easily discriminated (Figure 2 and Figure 3). In addition, both of the model evaluation parameters (R2Y and Q2Y) were about 1.0, and Q2Y was less than R2Y. This indicated that the model was not over-fitted, the data were repetitive, and the model was reliable.

**Figure 4:**
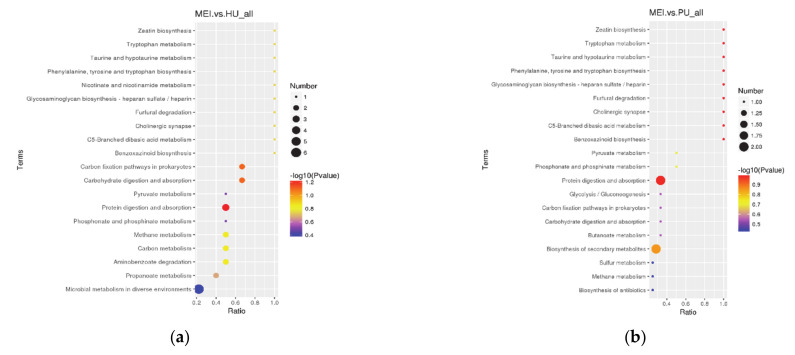

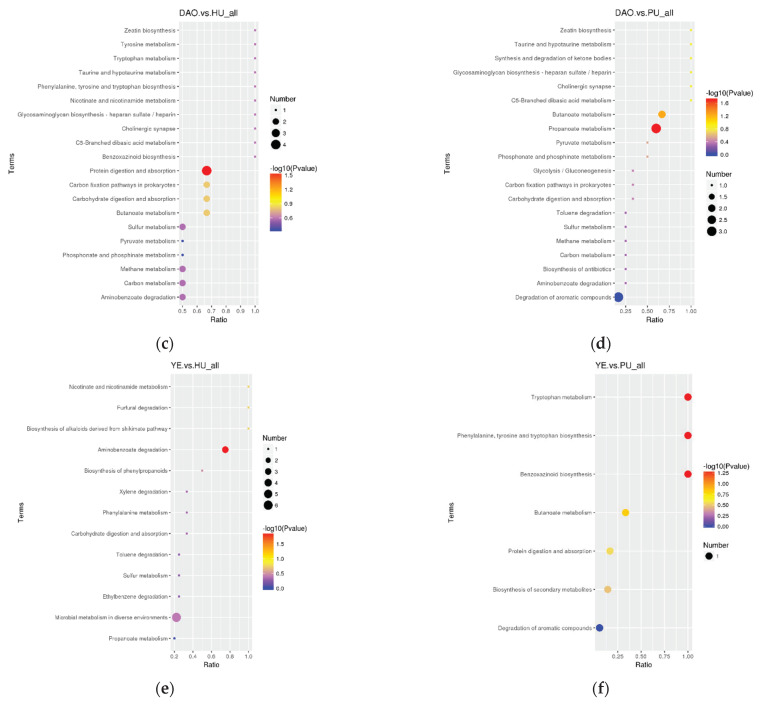
(**a**) The KEGG pathway that the significant differential metabolites take part in Meixiangzhan 2 vs. Huanghuazhan. (**b**) The KEGG pathway that the significant differential metabolites take part in Meixiangzhan 2 vs. Yanfeng 47. (**c**) The KEGG pathway that the significant differential metabolites take part in Daohuaxiang 2 vs. Huanghuazhan. (**d**) The KEGG pathway that the significant differential metabolites take part in Daohuaxiang 2 vs. Yanfeng 47. (**e**) The KEGG pathway that the significant differential metabolites take part in Yexiangyoulisi vs. Huanghuazhan. (**f**) The KEGG pathway that the significant differential metabolites take part in Yexiangyoulisi vs. Yanfeng 47. In these figures, the abscissa is x/y (i.e., the number of differential metabolites in the corresponding metabolic pathway divided by the total number of identified metabolites in the pathway). The higher the value on the abscissa, the higher the degree of differential metabolite enrichment in the corresponding pathway. Dot color represents the *p*-value of the hypergeometric test; smaller values reflect increased test reliability and greater statistical significance. The size of the dot represents the number of differential metabolites in the corresponding pathway; larger numbers indicate that more differential metabolites were identified in the corresponding pathway.

Comparison of Meixiangzhan 2 and Daohuaxiang 2 yielded 125 differential metabolites; comparison of Yexiangyoulisi and Daohuaxiang 2 yielded 129 differential metabolites; comparison of Yexiangyoulisi and Meixiangzhan 2 yielded 130 differential metabolites; comparison of Meixiangzhan 2 and Huanghuazhan yielded 155 differential metabolites; comparison of Daohuaxiang 2 and Huanghuazhan yielded 136 differential metabolites; comparison of Yexiangyoulisi and Huanghuazhan yielded 149 differential metabolites; comparison of Meixiangzhan 2 and Yanfeng 47 yielded 156 differential metabolites; comparison of Daohuaxiang 2 and Yanfeng 47 yielded 135 differential metabolites; and comparison of Yexiangyoulisi and Yanfeng 47 yielded 137 differential metabolites. The 30 metabolites that differed most significantly across all pairwise comparisons were used to screen key volatiles that distinguish aromatic rice from non-aromatic rice varieties (Table 2). The KEGG pathways associated with these 30 differential metabolites are shown in Figure 2 and Figure 4.

#### 2.2.1. Variation in Metabolic VOCs among Aromatic Rice Varieties

The results showed that the three aromatic rice varieties differed greatly (Figure 2a–c), that is, 10 metabolites distinguished Meixiangzhan 2 from the other varieties: (2-methyloctyl)benzene, 2,2-dichloroethanol, indole, 2,4,6-trimethylpyridine, 2-(octyloxy) ethanol, succinic acid but-3-yn-2-yl 2-methylpent-3-yl ester, 2-ethylheptanoic acid, 3-butene-1,2-diol, dibutoxymethane and 2-octen-1-ol (Table 2). Similarly, 11 differential metabolites were identified between Daohuaxiang 2 and other two aromatic rice varieties: butyl benzoate, DL-2-phenyl-1,2-propanediol, 2-acetyl-1-pyrroline, 1,3-dichloro-2-methyl benzene, sulfurous acid dodecyl pentyl ester, 2-(1-cyclopent-1-enyl-1-methylethyl)cyclopentanone, 2-methylpropanoic acid 3-hydroxy-2,2,4-trimethylpentyl ester, 6,6-dimethyl-cyclohex-2-en-1-ol, 2-bromocycloheptanone, 3,8-dihydroxy-3,4-dihydronaphthalen-1(2*H*)-one, and 1-(1*H*-pyrrol-2-yl)ethanone (Table 2). Finally, Yexiangyoulisi was distinguished from the other varieties by the metabolites (1*S*-*exo*)- 2-methyl-3-methylene-2-(4-methyl-3-pentenyl)bicyclo[2.2.1]heptane, 2-butyl-2-octenal, 1-ethenylaziridine, 2-methyl- 2-octen-4-ol, and 2-cyclohexylpiperidine (Table 2). No shared metabolites were identified among three aromatic rice varieties. Biosynthesis of secondary metabolites, protein digestion and absorption, and toluene degradation were the main pathways associated with the significantly different metabolites between Yexiangyoulisi and Daohuaxiang 2 (Figure 2d), while the metabolites that differed significantly between Meixiangzhan 2 and Daohuaxiang 2 were mainly associated with the biosynthesis of secondary metabolites, protein digestion and absorption, and aminobenzoate degradation (Figure 2e). The metabolites that differed significantly between Yexiangyoulisi and Meixiangzhan 2 were mainly associated with the biosynthesis of secondary metabolites, protein digestion and absorption, and ethylbenzene degradation (Figure 2f).

In general, the metabolites that differed significantly among the three aromatic rice varieties were associated with the biosynthesis of secondary metabolites and with protein digestion and absorption. The differences in the biosynthesis of secondary metabolites among the three kinds of rice grains were closely related to characteristics of the growth environment, such as climate, soil conditions, and altitude [1]. The geography and climate of the regions producing the three aromatic rice varieties used in this study differ substantially. Guangdong province has a warm climate, sufficient sunshine, abundant rainfall, and high levels of organic compounds in the soil [23]. Water can promote the synthesis of organic acids; these metabolically active solutes participate in osmotic adjustment and help to balance excess cations in plants [24]. Therefore, organic acids and their derivatives such as succinic acid but-3-yn-2-yl 2-methylpent-3-yl ester and heptanoic acid 2-ethyl were accumulated in Meixiangzhan 2. Additionally, 2,2-dichloroethanol, and 2-(octyloxy)ethanol, could be metabolites of dichlorodiphenyltrichloroethane (DDT) [25]. Daohuaxiang 2 is planted in Wuchang, Heilongjiang province, which is the most famous aromatic rice-growing area in China [4]. The soil in this region is mainly sandy loam and meadow soil, with abundant sunshine and widespread irrigation systems [6]. The 2-AP content of Daohuaxiang 2 was much greater than the 2-AP contents of the other aromatic rice varieties (Table 1). Higher soil nitrogen levels increase 1-proline content, which is the precursor of 2-AP; thus, aromatic rice from this region has a strong aroma [10,26]. Guangxi province has a warm climate, abundant rainfall, and moderate sunshine [27]. Due to the reduced sunshine exposure, Yexiangyoulisi does not accumulate as many secondary metabolites as other varieties, instead accumulating aromatic compounds with a benzene ring, such as (1*S-exo*)-2-methyl-3-methylene-2-(4-methyl-3-pentenyl)bicyclo[2.2.1]heptane, 2-butyl-2-octenal and 2-methyl-2-octen-4-ol, In addition, because aromatic rice is vulnerable to diseases and insect pests, a variety of agricultural chemicals, such as fertilizers and growth regulators, have been used in the cultivation of aromatic rice. It has been reported that manganese (Mn) application significantly increased 2-AP content in Meixiangzhan and Nongxiang 18, possibly due to the increased activity of enzymes involved in the formation of 2-AP [28].

#### 2.2.2. Variations in Metabolic VOCs between Aromatic and Non-Aromatic Rice

The rice sample data were repetitive, and the PLS-DA model data were reliable (Figure 3). There was a significant difference between aromatic and non-aromatic rice. Three shared metabolites were significantly differentially abundant between the non-aromatic variety Huanghuazhan and all three aromatic varieties (Meixiangzhan 2, Daohuaxiang 2, and Yexiangyoulisi): *trans-*verbenyl caprate, 1,3-dimethoxybenzene and 2-hexenal (Table 3). The metabolites significantly differentially abundant between Meizhanxiang 2 and Huanghuazhan were mainly associated with propanoate metabolism and with carbohydrate digestion and absorption (Figure 4a), while the metabolites significantly differentially abundant between Daohuaxiang 2 and Huanghuazhan were mainly associated with aminobenzoate degradation, carbon metabolism, methane metabolism, and sulfur metabolism (Figure 4c). Finally, the metabolites significantly differentially abundant between Yexiangyoulisi and Huanghuazhan were mainly associated with the propanoate metabolism and aminobenzoate degradation pathways (Figure 4e). Therefore, the metabolites that were significantly differentially abundant between Huanghuazhan rice and aromatic varieties were mainly associated with aminobenzoate degradation and with carbohydrate digestion and absorption.

Seven differentially abundant metabolites were significantly differentially abundant between the non-aromatic variety Yanfeng 47 and all three aromatic varieties (Meixiangzhan 2, Daohuaxiang 2, and Yexiangyoulisi): 2-isopropyl-5-methyl-9-methylenebicyclo[4.4.0]dec-1-ene, dodecamethylcyclohexasiloxane, 1-ethyl-5-methylcyclopentene, 1,2-dimethoxybenzene, 5-methyl-2-(1-methylethyl)-2-cyclohexen-1-one, 2-ethyl-2-(hydroxy-methyl)-1,3-propanediol and 5-ethyl-2-decen-4-one (Table 3). The primary pathway associated with the differentially abundant metabolites between Meizhanxiang 2 and Yanfeng 47 was the biosynthesis of secondary metabolites (Figure 4b); The primary pathway associated with the differentially abundant metabolites between Daohuaxiang 2 and Yanfeng 47 was the degradation of aromatic compounds (Figure 4d); and the primary pathways associated with the differentially abundant metabolites between Yexiangyoulisi and Yanfeng 47 were the degradation of aromatic compounds and the biosynthesis of secondary metabolites (Figure 4f). Thus, the significantly differentially abundant metabolites between Yanfeng 47 and the aromatic rice varieties were mainly associated with the degradation of aromatic compounds and the biosynthesis of secondary metabolites.

The main metabolites that were differentially abundant between Meixiangzhan 2 and the non-aromatic rice grains were acetoin, (2-methyloctyl)benzene, 2,6,7-trimethyldecane, 2,2-dichloroethanol, 1-ethenylaziridine, *trans*-2-(2-propynyloxy)cyclopentanol and acetic acid butyl ester (Table 2). The main metabolites that were differentially abundant between Daohuaxiang 2 and the non-aromatic rice grains were 2-AP, acetoin, 2-isopropyl-5-methyl-9-methylenebicyclo[4.4.0]dec-1-ene, 2-methylfuran, 2-methyldecane, 2-butyl-2-octenal, 2-hexenal, and 3-nonen-2-one (Table 2). The main metabolites that were differentially abundant between Yexiangyoulisi and the non-aromatic rice grain were 2-isopropyl-5-methyl-9-methylenebicyclo[4.4.0]dec-1-ene, 2-methylfuran, 2-hexenal, acetic acid butyl ester, 3-butylpyridine-1-oxide, 6-methyl-3-heptanone, and 2-ethyl-2-(hydroxymethyl)- 1,3-propanediol (Table 2).

Overall, the metabolites with the highest abundances in non-aromatic rice were 1,3-dimethoxybenzene, 1,2-dimethoxybenzene, and 5-methyl-2-(1-methylethyl)-2-cyclohexen-1-one (Table 3). The pathways of 1,3-dimethoxybenzene and 1,2-dimethoxybenzene are related to benzoate degradation and degradation of aromatic compounds [16]. 5-Methyl-2-(1-methylethyl)-2-cyclohexen-1-one is an aromatic compounds usually found in herbal essential oils or Chinese medicines [29] and its pathway is related to the degradation of aromatic compounds [16]. The metabolites with the highest abundances in aromatic rice were 2-hexenal, 2-isopropyl-5-methyl-9-methylenebicyclo[4.4.0]dec-1-ene, 2-ethyl-2-(hydroxymethyl)-1,3-propanediol and 5-ethyl-2-decen-4-one. Hexenal was considered as a marker compound to distinguish aromatic rice from non-aromatic rice varieties [3,30]. In this study, the content of 2-hexenal in three aromatic rice were much greater than the content of 2-hexenal in the non-aromatic rice varieties, moreover the 2-hexenal content of Daohuaxiang 2 was 38 times higher than the 2-hexenal contents of non-aromatic rice varieties (Table 3). (*E*)- 2-hexenal is produced by the lipoxygenase pathway and plays an important role in plant defense and protects the microbial proliferation in wounded areas [31]. It induces a significant increase in some membrane related fatty acids, including linear and branched fatty acids and unsaturated fatty acids, and releases free fatty acids [31]. In addition, (E)-2-hexenal has significant antibacterial activity against food spoilage and pathogenic microbial species [31]. 2-Isopropyl-5-methyl-9-methylenebicyclo[4.4.0]dec-1-ene is also an aromatic compound often found in plant essential oils and those essential oils possessed stronger repellency activity against pests [32]. 2-Ethyl-2-(hydroxymethyl)-1,3-propanediol and 5-ethyl-2-decen-4-one are produced by the fatty acids pathway and butanoate metabolism, respectively [16]. The metabolites that differed significantly between the aromatic and non-aromatic rice varieties were associated with the degradation of aromatic compounds, protein digestion and absorption, carbohydrate digestion and absorption, and the biosynthesis of secondary metabolites. VOC metabolites accumulated in aromatic rice varieties were formed mainly from carbohydrates (i.e., terpenes and furanones), fatty acids (i.e., aldehydes and alcohols produced by lipoxygenase or α- and β-oxidases), and amino acids (acids, alcohols, aldehydes, esters, lactones, flavor molecules containing N and S, benzene, and phenylpropane compounds) [11]. These compounds are positively related to aroma traits and nutritional qualities [33]. Moreover, secondary metabolites associated with biosynthesis of secondary metabolites pathway have obvious impacts on rice quality (nutritional and appearance qualities) [33].

Analysis of the main metabolic VOCs that differ among the five rice varieties identified certain characteristics that distinguish Meixiangzhan 2 and Daohuaxiang 2. Specifically, 2-AP can be used as marker metabolic to differentiate Daohuaxiang 2 from other rice varieties (Table 2). Consistent with this, 2-AP was identified as a marker of rice produced in Wuchang, China [3]. Acetoin can be used as a marker for the identification of Meixiangzhan 2 (Figure 1c). Acetoin is synthesized in trace amounts under certain conditions in some plants and animals [22]. For example, a pathway with acetoin as a key metabolite was proposed to explain acetaldehyde detoxication mechanisms in mammals, including humans [22]. The pathway of acetoin was associated with C5-branched dibasic acid metabolism and butanoate metabolism [16]. In this study, high levels of acetoin were found in the aromatic rice varieties, which may be related to characteristics of the planting environment in China, such as soil type.

## 3. Materials and Methods

### 3.1. Sample Preparation

Three representative aromatic rice varieties (Meixiangzhan 2, Daohuaxiang 2, and Yexiangyoulisi) were collected in Guangdong, Heilongjiang, and Guangxi provinces, respectively. Two non-aromatic rice varieties (Huanghuazhan and Yanfeng 47), which were used as controls, were collected in Hubei and Liaoning provinces, respectively. Meixiangzhan 2 *(Oryza sativa* L. subsp. *indica*) is a hybrid rice of Lemont/Fengaozhan; Yexiangyoulisi (*Oryza sativa* L. subsp. *indica*) is a hybrid rice of Yexiang A/R Lisi; Huanghuazhan (*Oryza sativa* L. subsp. *indica*) is a hybrid rice of Huangxinzhan/Fenghuazhan; Daohuaxiang 2 (*Oryza sativa L*. subsp. *japonica*) is a hybrid rice of Wuyoudao 1; and Yanfeng 47 (*Oryza sativa* L. subsp. *japonica*) is a hybrid rice of AB005S/Fengjin+Lingjing 5. The data comes from China rice data center.

All of the rice samples used in this study were newly harvested, unhulled rice grains. All rice grains were obtained from recognized local Chinese vendors and were stored in closed containers respectively in the 4 °C before being used for analysis. All rice grains were harvested no more than 1 month before being used. The fatty acids value of the Meixiangzhan 2, Yexiangyoulisi, and Huanghuazhan were 10.4 mg/100 g, 12.2 mg/100 g and 12.8 mg/100 g, respectively. The fatty acids value of the Daohuaxiang 2 and Yanfeng 47 were 6.7 mg/100 g and 8.2 mg/100 g, respectively. According to the “guidelines for evaluation of paddy storage charter” (GB/T 20569-2006), the fatty acids value of all rice grains were in a suitable range. Each variety was represented by five samples. Samples were analyzed over a period of three weeks.

### 3.2. Metabolite Extraction

VOC metabolites were extracted from each rice sample using HS-SPME as follows: 100 mg of the rice sample was transferred into a 20 mL headspace bottle, and 10 μL of 2-octanol (>99.5%, TCI Chemical, Shanghai, China,(10 mg/L stock in dH_2_O) was added to the headspace bottle as the internal standard; the automatic heating incubator was preheated for 15 min and then extracted at 60 °C for 30 min using an SPME fiber (DVB/CAR/PDMS, 2 cm, 50/30 µm; Supelco, Bellefonte, PA, USA). For every five samples, an empty vial analysis was performed to check for any carryover.

### 3.3. GC-MS Analysis

The fiber containing the extracted VOC metabolites was decomposed in a splitless injector at 250 °C for 4 min and analyzed using GC-MS with DB-Wax (30 m × 250 μm × 0.25 μm). GC-MS analysis was performed using an Agilent 7890 gas chromatograph system (Agilent Technologies, Santa Clara, CA, USA) coupled with a 5977B mass spectrometer (Agilent Technologies). Helium was used as the carrier gas, the front inlet purge flow was 3 mL/min, and the gas flow rate through the column was 1 mL/min. The initial temperature was held at 40 °C for 4 min, raised to 245 °C at a rate of 5 °C/min, and finally held at 245 °C for 5 min. The injection temperature, transfer line temperature, ion source temperature, and quad temperature were 250 °C, 250 °C, 230 °C, and 150 °C, respectively. The energy was −70 eV in electron impact mode. The mass spectrometry data were acquired in scan mode at an *m*/*z* range of 20–500 and a solvent delay of 0 min. All rice samples were mixed in equal amounts to generate a quality control (QC) sample, which was used to calibrate the GC-MS system and evaluate system stability throughout the experiment.

The raw data were preprocessed using Chroma TOF 4.3X (LECO Corporation, Saint Joseph, MI, USA). The data were first simply screened based on retention time (RT) and mass-to-charge ratio (*m*/*z*). Then, the exact molecular weight of each compound was determined based on the mass-to-charge ratio in the extracted ion chromatogram (XIC) diagram. The VOC metabolites in all rice samples were identified by matching the fragment ion or collision energy of each compound to an entry in the National Institute of Standards and Technology (NIST) database. The maximum permitted tolerance for relative ion intensities were ±5%. Deconvolution and integral calculus were performed on the spectra of the experimental samples. The peak area of each characteristic peak represented the relative abundances of a compound. The total peak area was used to normalize the quantitative results, and finally the quantitative results of the data were obtained.

### 3.4. Multivariate Metabolite Analysis

Principal components analysis (PCA) and partial least squares discriminant analysis (PLS-DA) were used to identify differences in metabolites among rice varieties. All pairwise comparisons: Meixiangzhan 2 vs. Daohuaxiang 2, Meixiangzhan 2 vs. Huanghuazhan, Meixiangzhan 2 vs. Yanfeng 47, Daohuaxiang 2 vs. Huanghuazhan, Daohuaxiang 2 vs. Yanfeng 47, Yexiangyoulisi vs. Daohuaxiang 2, Yexiangyoulisi vs. Huanghuazhan, Yexiangyoulisi vs. Meixiangzhan 2, and Yexiangyoulisi vs. Yanfeng 47. PLS-DA is a supervised statistical method in which partial least squares regressions are used to establish a relationship model between metabolite expression and sample category in order to predict sample category based on metabolite expression [34]. A PLS-DA model was established for each compared group, and the model evaluation parameters (R2 and Q2) were obtained using a seven-fold cross validation. The closer R2 and Q2 are to 1, the more stable and reliable the model is [11]. In addition, when Q2 was less than R2 and the y-intercept of Q2 was less than 0, the model was not over-fitted and the model was reliable. The variable importance in the projection (VIP) value of the first principal component of the PLS-DA model was used to represent the relative contribution of metabolite differences among groups. Fold change (FC), which was equivalent to the ratio of the mean quantitative values of the metabolites in the two compared groups, combined with the *p*-value of the *t*-test were employed to screen the differentially expressed metabolites and reduce the possibility of false positives. The threshold values used identify the differentially expressed metabolites were VIP > 1.0 and FC > 1.5 or FC < 0.667 with a *p*-value < 0.05. The Kyoto Encyclopedia of Genes and Genomes (KEGG) database, which is the most well-known public pathway database, was used to determine the most important metabolic pathways associated with the differential metabolites among varieties.

## 4. Conclusions

The VOCs metabolites of five rice grain varieties were identified and analyzed by HS-SPME-GC-MS based on an untargeted metabolomics approach, and PCA analysis and a PLS-DA model were used to clearly distinguish the aromatic and non-aromatic rice cultivars. The results showed that metabolomics analysis of rice grain could be well examine aroma trait related to rice authentication.

Accumulated volatile metabolites differed significantly between three aromatic rice (Daohuaxiang 2, Meixiangzha 2, and Yexiangyoulisi) and non-aromatic rice varieties. 2-Acetyl-1-pyrroline, acetoin and 2-hexenal were the marker metabolites for aromatic rice varieties. The metabolites that differed significantly among these aromatic rice varieties were associated with the biosynthesis of secondary metabolites and with protein digestion and absorption. This was related to the soil and climate conditions of their planting area. There were also obvious differences in metabolite accumulation between the aromatic and non-aromatic rice varieties; these significantly differentially abundant metabolites were associated with the degradation of aromatic compounds, protein digestion and absorption, carbohydrate digestion and absorption, and the biosynthesis of secondary metabolites. This makes aromatic rice superior to non-aromatic rice in both aroma traits and nutritional qualities. Finally, the establishment of libraries of aromatic rice from different regions will provide the basis for the authenticity identification and standard formulation of aromatic rice in the future.

## Figures and Tables

**Table 1:** The selected VOC metabolites with their relative abundances across all the cultivars.

RT [min]	Compounds	DAO	MEI	YE	HU	PU
	Shared metabolites					
15.29	1-Hexanol	1.72 × 10^8^	96,048,970	1.13 × 10^8^	24,102,107	45,055,543
3.78	Fluoromethyloxirane	36,614,040	23,305,508	44,202,528	28,416,649	1.45 × 10^8^
12.37	1-Pentanol	37,980,623	30,586,851	25,157,322	6,633,422	7,772,666
9.28	1-Butanol	26,388,511	13,311,345	15,000,191	8,151,245	3,133,619
1.73	Ethyl ether	27,745,422	25,783,629	22,836,831	24,493,908	33,315,764
2.36	Acetone	18,903,094	14,901,508	10,460,835	7,760,489	5,174,855
7.15	Hexanal	16,299,306	27,805,740	24,085,922	5,099,126	7,709,785
17.83	Acetic acid	15,818,963	36,842,037	11238248	2,015,046	5,899,140
17.87	1-Octen-3-ol	14,891,769	13,388,022	15,550,836	9,818,053	25,329,453
9.7	1-Penten-3-ol	12,473,204	8,297,892	8,223,888	2,972,998	4,730,423
23.32	2-Pentylfuran	12,281,335	9,644,697	11,429,015	3,493,209	5,800,664
18.03	1-Heptanol	5,921,051	7,433,490	6,080,963	4,442,551	6,035,516
42.15	Dibutyl phthalate	4,606,760	3,567,330	1,983,646	1,391,658	3,006,027
27.4	Hexanoic acid	2,799,541	3,840,783	3,387,139	4,241,496	1,079,895
26.14	2,4-Dimethylbenzaldehyde	4,469,583	8,813,779	10,020,634	3,439,346	3,472,676
10.21	Decamethylcyclopentasiloxane,	3,331,658	3,697,400	12,829,422	2,784,283	3,643,312
	Dimethylsilanediol					
	Important metabolites in Mei					
23.08	2,2-Dichloroethanol	1,224,567	11,1523,95	1,224,567	36,892,075	42,942,869
21.63	3-Methylbutanoic acid	63,498.64	4,545,779	16,655.63	69,413.39	33,309.38
23.52	Pentanoic acid	1,359,020	1,983,710	1,217,325	1,964,045	779,162.5
25.12	Nonanal	1,160,505	1,628,053	860,797.6	994,269.9	301,766.9
16.25	5-Ethenyldihydro-5-methyl-2(3*H*)-furanone	822,909.4	2,818,383	2,225,003	534,010.8	619,826.6
	Important metabolites in Hu					
22.96	*n*-Butyl ether	2,312,773	3,751,675	448,611	929,572.9	634,660.8
4.34	Indole	404,411.8	323,469.5	346,924.8	2,304,147	147,699.5
37.99	1,2,4,5-Tetramethylbenzene	361,177.9	33,235.97	412,467.8	1,828,698	259,974.3
16.95	(Z)-2-Hexen-1-ol	1,300,407	1,031,014	1,357,945	2,231,575	1,244,037
	Important metabolites in Pu					
16.67	3-Isopropoxy-1,1,1,7,7,7-hexamethyl-3,5,5-tris(trimethylsiloxy)tetrasiloxane	1,782,711	613,313.8	1,060,169	282,098.5	282,098.7
19.52	2,4,6-Trimethyldecane	447,896.4	454,443.5	464,899.2	437,090.2	18,992,198
6.06	2,6-Dimethylnonane-	207,859.7	344,146.2	287,853.3	286,306.8	3,265,933
17.62	2-Furanmethanol, 5-ethenyltetrahydro,5-trimethyl-	2,878,806	1,420,566	824,133.5	1,205,407	2,184,705
16.42	3-Octanol	1,906,009	798,094.8	1,202,779	759,883.7	1,993,274
27.74	2,2,4-Trimethyl-1,3-pentanediol diisobutyrate	1,607,821	1,984,047	977,550.7	827,407.3	1,767,177
	Important metabolites in Dao					
8.24	2-Butanone	3,680,787	3,095,654	1,581,748	1,120,188	864,840.9
14.63	2-Acetyl-1-pyrroline	3,452,700	133,350.6	265,893.5	48,794.77	48,795.2
21.49	1-Methylcycloheptanol	3,180,146	1,081,452	1,261,950	638,954.6	1,807,368
	Important metabolites in Aromatic rice					
13.1	Acetoin	88,606,134	24,735,894	3,040,508	2,235,303	882,512.3
10.16	2-Heptanone	4,476,631	3,992,086	3,172,086	1,076,111	1,275,083

Dao stands for the Daohuaxiang 2variety, Ye stands for the Yexiangyoulisi variety, Mei stands for the Meixiangzhan 2 variety, Hu stands for the Huanghuazhan variety and Pu stands for the Yanfeng 47 variety.

**Table 2:** Selected differential metabolites across all pairwise comparisons.

Differential Metabolites	RT [min]	*p*-Value	VIP
MEI vs. DAO			
Butyl benzoate	27.34	2.51 × 10^−9^	4.71
2,2-Dichloroethanol	21.64	1.79 × 10^−11^	4.57
Sulfurous acid dodecyl pentyl ester	16.81	0.000306	3.57
2,6,7-Trimethyldecane	6.03	0.088075	3.54
2-Acetyl-1-pyrroline	14.64	1.66 × 10^−9^	3.46
1,3-Dichloro-2-methylbenzene	19.75	3.05 × 10^−7^	3.22
(*Z*)-2-Octen-1-ol	21.96	1.29 × 10^−6^	3.03
4,8-Dimethylundecane	10.76	0.122349	3.02
2,4,6-Trimethylpyridine	15.71	2.17 × 10^−7^	2.87
2-Bromocycloheptanone	17.66	1.40 × 10^−6^	2.86
DL-2-Phenyl-1,2-propanediol	25.13	2.44 × 10^−7^	2.69
2-(1-Cyclopent-1-enyl-1-methylethyl)cyclopentanone	25.27	0.006072	2.61
2-(Octyloxy)ethanol-	7.83	4.70 × 10^−6^	2.46
Indole	37.99	0.000225	2.41
Borane-methyl sulfide complex	2.04	2.04 × 10^−6^	2.34
Methyl isobutyl ketone	5.19	0.001067	2.27
Tetramethylpyrazine	18.45	0.195585	2.24
1,4-Diethoxybenzene	28.53	1.73 × 10^−5^	2.19
6,6-Dimethylcyclohex-2-en-1-ol	26.87	2.55 × 10^−6^	2.18
Succinic acid but-3-yn-2-yl 2-methylpent-3-yl ester	15.49	3.62 × 10^−6^	2.12
3-Methylpyridine	13.42	4.53 × 10^−7^	2.12
2-Ethylheptanoic acid	26.99	0.00476	2.10
1-Ethyl-3-methylbenzene	11.33	0.000117	2.08
1-(1*H*-Pyrrol-2-yl)-ethanone	29.47	3.45 × 10^−8^	2.05
Dibutoxymethane	11.28	2.11 × 10^−5^	2.03
3-Butene-1,2-diol	8.35	1.59 × 10^−6^	2.03
Furfural	17.95	8.58 × 10^−5^	2.02
3,8-Dihydroxy-3,4-dihydronaphthalen-1(2*H*)-one	38.62	1.33 × 10^−5^	1.98
4-Methylhexyl isobutyrate	9.18	6.59 × 10^−6^	1.97
MEI vs. HU			
(2-Methyloctyl)benzene	6.37	0.003302	6.53
2,6,7-Trimethyldecane	6.03	0.030641	5.37
2,2-Dichloroethanol	21.64	9.68 × 10^−5^	4.44
2-Butyl-2-octenal	23.12	5.16 × 10^−6^	4.15
Indole	37.99	7.70 × 10^−7^	3.58
Tetramethylpyrazine	18.45	0.017066	3.52
2-Isopropyl-5-methyl-9-methylenebicyclo[4.4.0]dec-1-ene	21.43	0.056959	3.49
*trans*-Verbenyl caprate	14.35	0.01666	3.31
Carbon monoxide	2.25	0.023602	3.14
1,2-Dimethoxybenzene	24.32	6.56 × 10^−6^	3.03
(1*S*-*exo*)-2-Methyl-3-methylene-2-(4-methyl-3-pentenyl)bicyclo[2.2.1]heptane	22.69	1.43 × 10^−5^	2.99
1,3-Dimethoxybenzene	24.80	2.90 × 10^−8^	2.76
Acetoin	13.11	3.12 × 10^−5^	2.76
2,4,6-Trimethylpyridine	15.71	2.17 × 10^−7^	2.40
(*E*)-2-Hexenal	11.16	0.002133	2.28
1-Ethyl-3-methylbenzene	11.33	5.30 × 10^−5^	2.26
1-Ethenylaziridine	5.45	1.55 × 10^−5^	2.25
*trans-*2-(2-propynyloxy)cyclopentanol	24.23	0.001288	2.16
6,10,14-Trimethyl-pentadecan-2-ol	26.30	2.04 × 10^−5^	2.15
Acetic acid	17.84	0.000139	2.00
Acetic acid butyl ester	6.97	3.34 × 10^−5^	1.98
(*Z*)-2-Octen-1-ol,	21.96	0.001148	1.97
3-Nonen-2-one	19.32	0.044049	1.95
Pentanal	4.54	0.00045	1.90
1-Ethyl-4-methylbenzene	13.05	0.000112	1.87
Propanoic acid butyl ester	8.96	5.78 × 10^−5^	1.87
5-Ethyl-2-decen-4-one	23.50	0.000312	1.85
1-(3,3-Dimethyloxiranyl)ethanone	13.61	0.004344	1.84
3-Methyl-6-ethyl-2,4-dioxadecane	21.00	1.83 × 10^−6^	1.84
3-Methyl-2-butenal-	10.58	1.01 × 10^−5^	1.84
Mei vs. PU			
(2-Methyloctyl)benzene	6.37	3.01 × 10^−8^	6.83
2,6,7-Trimethyldecane	6.03	0.048495	4.75
2,2-Dichloroethanol	21.64	0.000132	3.81
1,2-Dimethoxybenzene	24.32	5.19 × 10^−9^	3.79
2-Butyl-2-octenal	23.12	1.36 × 10^−7^	3.69
Tetramethylpyrazine	18.45	0.017066	3.35
2-Isopropyl-5-methyl-9-methylenebicyclo[4.4.0]dec-1-ene	21.43	0.044206	3.31
1-Ethyl-5-methylcyclopentene	4.59	1.84 × 10^−6^	3.14
4,8-Dimethylundecane	10.76	0.092829	3.04
Dodecamethylcyclohexasiloxane	15.17	1.00 × 10^−9^	3.01
3-Isopropoxy-1,1,1,7,7,7-hexamethyl-3,5,5-tris(trimethylsiloxy)tetrasiloxane	19.53	1.52 × 10^−8^	2.98
Acetoin	13.11	2.78 × 10^−6^	2.86
Propanoic acid butyl ester	8.96	0.000923	2.78
*trans*-2-(2-Propynyloxy)cyclopentanol	24.23	0.000126	2.49
n-Butylbenzene-	13.93	0.071259	2.45
2-Propenoic acid butyl ester	10.00	0.000325	2.32
(*Z*)-2-Octen-1-ol	21.96	6.04 × 10^−6^	2.28
1-Ethenylaziridine	5.45	6.06 × 10^−7^	2.26
Acetic acid butyl ester	6.97	1.03 × 10^−8^	2.22
3-Octen-2-one	16.60	0.000309	2.22
5-Oxotetrahydrofuran-2-carboxylic acid	21.35	0.00012	2.21
5-Ethyl-2-decen-4-one	23.50	3.86 × 10^−5^	2.21
6,10,14-Trimethyl-pentadecan-2-ol	26.30	1.48 × 10^−5^	2.14
5-Methyl-2-(1-methylethyl)-2-cyclohexen-1-one	23.68	0.000157	2.13
3-Nonen-2-one	19.32	0.036233	2.10
2-Ethyl-2-(hydroxymethyl)-1,3-propanediol	14.11	1.26 × 10^−8^	2.09
2,5-Dimethyl-2,4-hexadiene	5.92	1.18 × 10^−5^	2.03
Pentanoic acid	25.13	0.000287	1.98
Pentanal	4.54	3.83 × 10^−5^	1.97
2-Methyldecane	7.90	0.03382	1.88
DAO vs. HU			
2-Isopropyl-5-methyl-9-methylenebicyclo[4.4.0]dec-1-ene	21.43	0.021135	5.63
(1*S*-*exo*)-2-Methyl-3-methylene-2-(4-methyl-3-pentenyl)bicyclo[2.2.1]heptane	22.69	1.76 × 10^−6^	4.87
Butyl benzoate	27.34	5.09 × 10^−6^	4.46
2-Acetyl-1-pyrroline	14.64	3.82 × 10^−7^	4.38
4,8-Dimethylundecane	10.76	0.127631	3.90
*trans*-Verbenyl caprate	14.35	0.016663	3.87
2-Butyl-2-octenal	23.12	5.95 × 10^−5^	3.24
1,3-Dimethoxybenzene	24.80	2.90 × 10^−8^	3.23
Dibutoxymethane	11.28	3.72 × 10^−8^	3.06
1-Ethenylaziridine	5.45	1.63 × 10^−5^	3.05
Carbon monoxide	2.25	0.041224	3.05
Sulfurous acid dodecyl pentyl ester	16.81	0.000566	3.03
(*E*)-2-Hexenal	11.16	0.001103	3.02
DL-2-Phenyl-1,2-propanediol	25.13	0.000277	3.02
1-(1*H*-Pyrrol-2-yl)ethanone	29.47	1.36 × 10^−6^	2.97
2-Methyldecane	7.90	0.005357	2.96
2-Bromocycloheptanone	17.66	1.40 × 10^−6^	2.79
2,6,7-Trimethyldecane	6.03	0.289158	2.79
2-Methylpropanoic acid 3-hydroxy-2,2,4-trimethylpentyl ester	27.52	2.16 × 10^−7^	2.73
Acetoin	13.11	0.005829	2.62
3-Nonen-2-one	19.32	0.030047	2.58
Cyclopropanecarboxylic acid oct-3-en-2-yl ester	13.71	0.01617	2.42
4-*tert*-Butoxystyrene	37.17	3.46 × 10^−7^	2.32
2-Methyl-2-octen-4-ol	26.50	2.27 × 10^−5^	2.29
Aniline	11.27	7.85 × 10^−7^	2.29
2-Methylfuran,	23.33	2.14 × 10^−8^	2.27
N-(1,1-Dimethylprop-2-ynyl)-acetamide	18.00	0.000164	2.21
4-Ethyl-4*H*-1,2,4-triazole	4.98	0.006118	2.16
3-Methyl-2-butenal	10.58	6.18 × 10^−6^	2.13
1,3-Dichloro-2-methylbenzene	19.75	9.93 × 10^−10^	2.08
DAO vs. PU			
4,8-Dimethylundecane	10.76	0.00596	6.23
2-Isopropyl-5-methyl-9-methylenebicyclo[4.4.0]dec-1-ene	21.43	0.015716	5.23
2-Acetyl-1-pyrroline	14.64	3.82 × 10^−7^	4.09
Butyl benzoate	27.34	4.87 × 10^−5^	4.08
3-Isopropoxy-1,1,1,7,7,7-hexamethyl-3,5,5-tris(trimethylsiloxy)tetrasiloxane	19.53	2.65 × 10^−9^	3.58
Dodecamethylcyclohexasiloxane	15.17	1.24 × 10^−10^	3.46
1-Ethyl-5-methylcyclopentene	4.59	2.14 × 10^−7^	3.16
Sulfurous acid dodecyl pentyl ester	16.81	0.000194	3.16
1-Ethenylaziridine	5.45	6.88 × 10^−8^	2.99
2-Methyldecane	7.90	0.002899	2.97
1-(1*H*-pyrrol-2-yl)ethanone	29.47	1.36 × 10^−6^	2.77
2-Butyl-2-octenal	23.12	4.04 × 10^−6^	2.74
Acetoin	13.11	0.003984	2.73
3-Nonen-2-one	19.32	0.025678	2.71
1,3-Dichloro-2-methylbenzene	19.75	7.24 × 10^−5^	2.65
1,2-Dimethoxybenzene	24.32	5.15 × 10^−7^	2.63
2-Bromocycloheptanone	17.66	1.40 × 10^−6^	2.61
2-Methylfuran	23.33	1.03 × 10^−9^	2.56
2,4,6-Trimethyldecane	6.06	1.34 × 10^−9^	2.52
5-Methyl-2-(1-methylethyl)-2-Cyclohexen-1-one	23.68	2.08 × 10^−5^	2.51
1-Butanol	9.29	4.94 × 10^−8^	2.42
2-Ethyl-2-(hydroxymethyl)-1,3-propanediol	14.11	1.56 × 10^−5^	2.40
2,5-Dimethyl-2,4-hexadiene	5.92	1.88 × 10^−6^	2.40
2-(1-Cyclopent-1-enyl-1-methylethyl)cyclopentanone	25.27	0.006072	2.39
4-(5-Methyl-2-furanyl)-2-butanone	23.44	3.10 × 10^−5^	2.34
3,3-Dimethylcyclohexanol	19.28	0.000241	2.33
*n*-Butylbenzene	13.93	0.129919	2.28
(*E*)-2-Hexenal	11.16	0.006958	2.24
5-Ethyl-2-decen-4-one	23.50	3.04 × 10^−5^	2.22
2-Propenoic acid butyl ester	10.00	0.000258	2.19
YE vs. DAO			
Butyl benzoate	27.34	2.51 × 10^−9^	5.92
(1*S*-*exo*)-2-Methyl-3-methylene-2-(4-methyl-3-pentenyl)bicyclo[2.2.1]heptane	22.69	2.61 × 10^−7^	4.76
DL-2-Phenyl-1,2-propanediol	25.13	4.35 × 10^−5^	4.24
2-Acetyl-1-pyrroline	14.64	0.003009	4.14
1,3-Dichloro-2-methylbenzene	19.75	3.05 × 10^−7^	4.05
Sulfurous acid dodecyl pentyl ester	16.81	0.000667	3.70
2-Butyl-2-octenal	23.12	0.002661	3.55
2-Methyldecane	7.90	0.006932	3.36
2-(1-Cyclopent-1-enyl-1-methylethyl)cyclopentanone	25.27	0.006072	3.28
Dimethysilanediol,	23.08	0.053691	2.99
Propanoic acid, 2-methyl-, 3-hydroxy-2,2,4-trimethylpentyl ester	27.52	4.85 × 10^−7^	2.95
Aziridine, 1-ethenyl-	5.45	0.000476	2.95
2-Octen-4-ol, 2-methyl-	26.50	2.27 × 10^−5^	2.94
Undecane, 4,8-dimethyl-	10.76	0.22725	2.82
6,6-Dimethyl-cyclohex-2-en-1-ol	26.87	4.96 × 10^−5^	2.55
3-Nonen-2-one	19.32	0.017839	2.55
2-Bromocycloheptanone	17.66	0.000474	2.55
2-Methyl-2-propenoic acid 4-formyl-2-methoxyphenyl ester	13.88	6.99 × 10^−7^	2.54
4-Ethyl-4*H*-1,2,4-triazole	4.98	1.12 × 10^−6^	2.51
3,8-Dihydroxy-3,4-dihydronaphthalen-1(2*H*)-one	38.62	1.33 × 10^−5^	2.49
1-(1*H*-Pyrrol-2-yl)ethanone	29.47	2.00 × 10^−7^	2.43
2-Cyclohexylpiperidine	23.57	0.000698	2.40
4,8-Dimethylnona-3,8-dien-2-one	22.76	6.88 × 10^−6^	2.34
4-*tert*-Butoxystyrene	37.17	0.000971	2.31
Methyl 8,11,14,17-eicosatetraenoate	24.46	0.005069	2.26
N-(1,1-Dimethyl-prop-2-ynyl)-acetamide	18.00	0.025511	2.22
Butanal	2.88	0.016879	2.21
(2-Methyloctyl)benzene	6.37	0.372951	2.21
1-*sec*-Butyl-3-nitro-4-amino-1,2,5-triazole 2-oxide	13.53	0.038085	2.20
Benzaldehyde	19.44	0.000244	2.09
YE vs. HU			
2-Isopropyl-5-methyl-9-methylenebicyclo[4.4.0]dec-1-ene	21.43	0.04846	5.67
*trans*-Verbenyl caprate	14.35	0.021689	4.56
2,6,7-Trimethyldecane	6.03	0.253466	4.02
Dibutoxymethane	11.28	2.49 × 10^−5^	3.84
1,3-Dimethoxybenzene	24.80	0.000178	3.79
(*E*)-2-Hexenal	11.16	0.002022	3.51
1,2-Dimethoxybenzene	24.32	8.75 × 10^−7^	3.25
1,5-Dimethyl-2-oxabicyclo[3.2.1]nonan-7-one	13.91	7.20 × 10^−7^	3.07
4-Chlorophenol	37.88	7.32 × 10^−7^	2.85
Hentriacontane	18.05	0.002675	2.84
*n*-Butylbenzene	13.93	0.022446	2.76
6-Methylhept-4-en-1-yl 2-methylbutanoate	15.88	0.000103	2.74
2-Propenoic acid butyl ester	10.00	1.54 × 10^−5^	2.70
Acetic acid butyl ester	6.97	7.49 × 10^−6^	2.68
5-Chloroguaiacol	33.81	0.000571	2.64
Carbon monoxide	2.25	0.234365	2.60
2-Ethyl-2-(hydroxymethyl)-1,3-propanediol	14.11	0.000948	2.53
2-Ethylfuran	4.03	7.34 × 10^−6^	2.50
*n*-Butyl ether	4.35	3.31 × 10^−6^	2.44
Furan, 2-methyl-	23.33	3.64 × 10^−7^	2.43
1-Ethyl-4-methylbenzene	13.05	7.33 × 10^−6^	2.43
3-Butylpyridine-1-oxide	20.20	3.01 × 10^−7^	2.36
4,8-Dimethylundecane	10.76	0.522126	2.35
(*E*)-4-Oxohex-2-enal	24.96	0.000801	2.33
Methyl 8,11,14,17-eicosatetraenoate	24.46	0.005068	2.32
(*R*)-(−)-2-Pentanol	8.62	0.01173	2.29
1-(3,3-Dimethyloxiranyl)ethanone	13.61	0.010545	2.23
N,N,2,2-Tetramethyl-1,3-propanediamine	1.86	0.07517	2.19
4-Methylundecane	9.39	0.010168	2.18
*cis*-2-(2-Pentenyl)furan	13.68	2.03 × 10^−6^	2.17
YE vs. MEI			
(2-Methyloctyl)benzene	6.37	0.01419	8.07
2,2-Dichloroethanol	21.64	8.96 × 10^−11^	5.78
2-Butyl-2-Octenal	23.12	0.000407	4.95
Indole	37.99	4.57 × 10^−5^	4.25
2,6,7-Trimethyldecane	6.03	0.104491	3.76
Tetramethylpyrazine	18.45	0.087738	3.46
*trans-*2-(2-Propynyloxy)cyclopentanol	24.23	6.94 × 10^−5^	3.38
2,4,6-Trimethylpyridine	15.71	2.17 × 10^−7^	3.17
2(3H)-Furanone, 5-ethenyldihydro-5-methyl-	22.96	1.18 × 10^−8^	2.66
(1*S*-*exo*)-2-Methyl-3-methylene-2-(4-methyl-3-pentenyl)bicyclo[2.2.1]heptane	22.69	0.000391	2.64
2-(Octyloxy)ethanol	7.83	1.18 × 10^−5^	2.56
2-Propylthiophene	16.71	0.003933	2.49
3,5,5-Trimethyl 2(5*H*)-furanone	20.85	1.26 × 10^−6^	2.42
Nonanoic acid ethyl ester	20.03	0.111112	2.39
2-Methyl-2-octen-4-ol	26.50	4.19 × 10^−5^	2.36
Succinic acid but-3-yn-2-yl 2-methylpent-3-yl ester	15.49	3.62 × 10^−6^	2.35
2-Ethylheptanoic acid	26.99	0.003497	2.34
N,N-2,2-Tetramethyl-1,3-propanediamine	1.86	0.028851	2.30
Acetoin	13.11	0.000493	2.27
3-Cyano-3-methyl-4-oxopentanamide	29.50	1.21 × 10^−5^	2.24
3-Butene-1,2-diol	8.35	6.70 × 10^−6^	2.22
1-Ethenylaziridine	5.45	0.000907	2.13
(*S*)- N,N-2-Trimethyl-2-[(2,2,3-trimethyl-1-pyrrolidinyl)oxy]-1-propanamine	24.34	3.86 × 10^−6^	2.12
1-Ethyl-5-methylcyclopentene	4.59	1.95 × 10^−7^	2.09
10-Methylnonadecane	8.20	5.06 × 10^−9^	2.08
Dibutoxymethane	11.28	2.33 × 10^−5^	2.08
1-(2-Furanyl)1-propanone	7.74	0.016649	2.07
2-Cyclohexylpiperidine	23.57	0.000879	2.04
(*Z*)-2-Octen-1-ol,	21.96	0.020305	2.02
Butanoic acid butyl ester	11.32	0.003751	2.02
YE vs. PU			
4,8-Dimethylundecane	10.76	0.055871	4.87
2-Isopropyl-5-methyl-9-methylenebicyclo[4.4.0]dec-1-ene	21.43	0.037269	4.63
Dodecamethylcyclohexasiloxane	15.17	2.01 × 10^−9^	4.23
3-Isopropoxy-1,1,1,7,7,7-hexamethyl-3,5,5-tris(trimethylsiloxy)tetrasiloxane	19.53	1.55 × 10^−9^	4.09
(1*S*-*exo*)-2-methyl-3-methylene-2-(4-methyl-3-pentenyl)bicyclo[2.2.1]heptane	22.69	2.61 × 10^−7^	4.00
1,2-Dimethoxybenzene-	24.32	6.97 × 10^−6^	3.86
2-Propenoic acid butyl ester	10.00	0.000127	3.26
*n*-Butylbenzene	13.93	0.071271	3.25
3-Butylpyridine-1-oxide	20.20	1.06 × 10^−8^	3.11
3,3-Dimethylcyclohexanol	19.28	2.22 × 10^−8^	3.06
2,6,7-Trimethyldecane	6.03	0.352155	2.85
Propanoic acid butyl ester	8.96	0.001745	2.81
2,4,6-Trimethyldecane	6.06	7.58 × 10^−8^	2.76
2-Ethyl-2-(hydroxymethyl)-1,3-propanediol	14.11	2.72 × 10^−6^	2.69
Indole	37.99	0.000491	2.66
Acetic acid butyl ester	6.97	1.66 × 10^−5^	2.64
DL-2-Phenyl-1,2-propanediol	25.13	3.78 × 10^−5^	2.64
Dimethylsilanediol	23.08	0.047035	2.62
1-*sec*-Butyl-3-nitro-4-amino-1,2,5-triazole 2-oxide	13.53	0.017105	2.58
2-Methylfuran	23.33	2.20 × 10^−9^	2.50
3-Octen-2-one	16.60	0.001749	2.48
Decanoic acid ethyl ester	22.49	0.016237	2.42
5-Methyl-2-(1-methylethyl)-2-cyclohexen-1-one	23.68	0.000895	2.37
N-(3,5-dihydroxyphenyl)acetamide,	30.19	0.000652	2.37
2,6-Dimethylnonane	5.40	6.76 × 10^−5^	2.28
Hentriacontane	18.05	0.01257	2.28
6-Methyl-3-heptanone	12.33	0.00116	2.22
Hexadecamethylcyclooctasiloxane	23.48	1.49 × 10^−6^	2.21
5-Ethyl-2-decen-4-one	23.50	0.000115	2.21
(*E*)-2-Hexenal	11.16	0.013029	2.19

VIP: variable importance in the projection; all pairwise comparisons: Meixiangzhan 2 vs. Daohuaxiang 2, Meixiangzhan 2 vs. Huanghuazhan, Meixiangzhan 2 vs. Yanfeng 47, Daohuaxiang 2 vs. Huanghuazhan, Daohuaxiang 2 vs. Yanfeng 47, Yexiangyoulisi vs. Daohuaxiang 2, Yexiangyoulisi vs. Huanghuazhan, Yexiangyoulisi vs. Meixiangzhan 2, and Yexiangyoulisi vs. Yanfeng 47.

**Table 3:** The significant different metabolites and their relative abundance between aromatic and non-aromatic rice varieties.

Different Metabolites	RT [min]	DAO	MEI	YE	HU	PU
*trans*-Verbenyl caprate	14.35	808.04	808.21	2880.04	808.47	808.536
1,3-Dimethoxybenzene	24.79	10,925.11	10,925.54	10,925.39	228,638	10,925.27
(*E*)-2-Hexenal	11.16	1,082,045	455,636.2	776,304.4	28,205.43	28,205.94
2-Isopropyl-5-methyl-9-methylenebicyclo[4.4.0]dec-1-ene	21.42	222,991.9	53,964.66	75,151.33	37,707.41	38,170.74
Dodecamethylcyclohexasiloxane	15.16	3,377,694	2,973,434	2,735,523	2,927,507	1.2 × 10^8^
1-Ethyl-5-methylcyclopentene	4.59	561,031.6	717,270.5	107,995	126,177.6	15,334.66
1,2-Dimethoxybenzene	24.32	14,742.91	2068.13	9001.32	62,189.44	179,016.1
5-Methyl-2-(1-methylethyl)-2-cyclohexen-1-one,	23.67	9476.75	9477.015	25,774.79	42,351.68	139,730.8
2-Ethyl-2-(hydroxymethyl)-1,3-propanediol	14.1	883,789.3	712,170	723,473.1	64,951.88	64,952.48
5-Ethyl-2-decen-4-one	23.49	193,215.2	213,350.9	129,560.4	33,279.31	15,463.01

Dao stands for the variety of Daohuaxiang 2, Ye stands for the variety of Yexiangyoulisi, Mei stands for the variety of Meixiangzhan 2, Hu stands for the variety of Huanghuazhan, and Pu stands for the variety of Yanfeng 47.

## Data Availability

The data presented in this study are available in article.
